# *HELICOBACTER PYLORI* BEYOND THE STOMACH: WHEN THE STORY CHANGES

**DOI:** 10.1590/S0004-2803.24612026-031

**Published:** 2026-07-20

**Authors:** Bruno Squárcio Fernandes SANCHES, Sandro Rodrigues CHAVES, Frederico Passos MARINHO, Guilherme Grossi Lopes CANÇADO, Daniel Antônio Albuquerque TERRA, Maria Clara Freitas COELHO, Luiz Gonzaga Vaz COELHO

**Affiliations:** 1 Núcleo Brasileiro para o Estudo do Helicobacter pylori e Microbiota, São Paulo, SP, Brasil.; 2 Private practice, Belo Horizonte, MG, Brasil.; 3 Rede Mater Dei de Saude, Belo Horizonte, MG, Brasil.; 4 Universidade Federal de Minas Gerais, Faculdade de Medicina, Programa de Pós-Graduação em Ciências do Adulto, Belo Horizonte, MG, Brasil.; 5 Universidade Federal de Minas Gerais, Hospital das Clínicas, Instituto Alfa de Gastroenterologia, Belo Horizonte, MG, Brasil.; 6 Hospital da Polícia Militar de Minas Gerais, Belo Horizonte, MG, Brasil.; 7 Faculdade de Ciências Médicas de Minas Gerais, Belo Horizonte, MG, Brasil.

**Keywords:** Helicobacter pylori, anemia, immune thrombocytopenia, colorectal neoplasms, cardiovascular diseases, nervous system diseases, Helicobacter pylori, anemia, trombocitopenia imune, neoplasia colorretal, doenças cardiovasculares, doenças do sistema nervoso

## Abstract

**Background:**

Helicobacter pylori (H. pylori) is well known as an etiological agent in several gastric diseases, including chronic gastritis, gastric and duodenal ulcers, gastric carcinoma, and lymphoma. Over the past decades, the understanding of its pathogenic role has evolved substantially, overcoming initial skepticism and establishing the bacterium as a major factor in gastroduodenal disease. However, epidemiological and experimental studies suggest that this chronic infection may also be associated with a variety of extragastric conditions.

**Objective:**

This review aims to evaluate the relationship between H. pylori infection and anemia, immune thrombocytopenic purpura, colorectal cancer, cardiovascular diseases, and neurological disorders.

**Methods:**

For each association, the most recent evidence and current recommendations regarding the diagnosis and treatment of H. pylori infection during the clinical course of these conditions were assessed.

**Conclusion:**

Further prospective studies are needed to clarify whether these associations represent causal relationships with clinical relevance or merely statistical correlations, and to better determine the impact of H. pylori infection on extragastric diseases.

## HIGHLIGHTS

• In this review, we reviewed and synthesized recent evidence addressing the association between Helicobacter pylori infection and a wide spectrum of extragastric conditions.

• Understanding broader pathogenic roles may impact clinical management.

## RESUMO

**Contexto:**

*Helicobacter pylori* (*H. pylori*) é amplamente reconhecido como agente etiológico de diversas doenças gástricas, incluindo gastrite crônica, úlceras gástricas e duodenais, carcinoma gástrico e linfoma. Ao longo das últimas décadas, a compreensão de seu papel patogênico evoluiu substancialmente, superando o ceticismo inicial e estabelecendo a bactéria como importante fator nas doenças gastroduodenais. No entanto, estudos epidemiológicos e experimentais sugerem que essa infecção crônica também pode estar associada a uma variedade de condições extra-gástricas.

**Objetivo:**

Esta revisão tem como objetivo avaliar a relação entre a infecção por *H. pylori* e anemia, púrpura trombocitopênica imune, câncer colorretal, doenças cardiovasculares e distúrbios neurológicos.

**Métodos:**

Para cada associação, foram analisadas as evidências mais recentes e as recomendações atuais quanto ao diagnóstico e tratamento da infecção por *H. pylori* durante o curso clínico dessas condições.

**Conclusão:**

Estudos prospectivos adicionais são necessários para esclarecer se essas associações representam relações causais com relevância clínica ou apenas correlações estatísticas, bem como para melhor determinar o impacto da infecção por *H. pylori* nas doenças extra-gástricas.

## INTRODUCTION

It is well established that, in addition to chronic gastritis, *H. pylori* infection is the main risk factor for gastric cancer. The hypothesis that certain microorganisms can cause diseases at sites distant from their primary site of infection by interfering with multiple biological processes has been increasingly explored in the context of *H. pylori*. This concise review highlights the most relevant, recently published studies on the association between *H. pylori* and hematologic disorders (iron deficiency anemia [IDA] and immune thrombocytopenia [ITP]), colorectal cancer (CRC), cardiovascular disease (CVD) (coronary artery disease and atherosclerosis), neurological conditions (Parkinson disease [PD], stroke, and multiple sclerosis), and dementia, including Alzheimer disease. The potential role of *H. pylori* in the various proposed pathogenic mechanisms is explored in detail. Herein, we review the initial findings of published therapeutic eradication trials and evaluate the potential role of eradication as a preventive strategy in specific clinical scenarios. New studies are eagerly awaited on this fascinating topic.

### Iron deficiency anemia

The link between unexplained IDA and *H. pylori* infection first emerged from pediatric case reports, which were later corroborated by population-based studies in adults and subsequent research^1^ ^-^ ^6^.

The pathophysiology is multifactorial^7^ ^-^ ^9^. The attachment of *H. pylori* to the gastric mucosa alters iron (Fe) homeostasis^7^ ^-^ ^9^. While blood loss due to mucosal lesions caused by *H. pylori* is relatively common in adults, it is very rare in children^7^. Corpus-predominant gastritis can decrease gastric acidity, thereby impairing both the reduction of Fe^3+^ to Fe^2+^ and the secretion of ascorbic acid, which are critical for intestinal iron absorption^10^. However, the corpus-predominant gastritis phenotype is less frequently observed with *H. pylori* infection, and it is even rarer in children. This indicates that decreased gastric acidity is likely not the primary driver in the pathogenesis of IDA^7^ ^,^ ^11^.

Gastric inflammation can lead to an increase in interleukin-1β (IL-1β), which correlates with decreased ferritin and hemoglobin (Hb) levels^12^. There may also be an elevation in IL-6, which, together with IL-1β, stimulates the production of hepcidin-a hormone primarily synthesized in the liver that inhibits intestinal iron absorption. This mechanism may explain why patients remain refractory to iron supplementation unless *H. pylori* is eradicated^7^ ^,^ ^9^.

Bacterial genetic polymorphisms leading to higher expression of vacuolating cytotoxin A (VacA), neutrophil-activating protein A (NapA), and sialic acid-binding adhesin (SabA) are also associated with IDA^7^ ^,^ ^9^.

Nevertheless, the primary mechanism seems to be direct competition for available iron between the host and *H. pylori*, as the bacterium has a high iron demand due to its rapid metabolism and turnover^7^ ^,^ ^9^. Building on Barabino’s seminal hypothesis, it has been revealed that certain strains possess iron-scavenging proteins, such as Fur (high affinity for Fe^3+^), FeoB (Fe^2+^), FecA (ferric dicitrate), and Pfr (non-heme ferritin). These proteins induce and perpetuate IDA as the bacterium upregulates their expression in iron-depleted environments^7^ ^,^ ^9^ ^,^ ^13^.

The increase in IL-1β, resulting from the upregulation of specific host genes in some infected individuals, is a host-dependent mechanism^7^. Other host-related factors involve increased iron requirements during specific life stages, such as childhood, adolescence (due to rapid growth), and pregnancy^7^.

A meta-analysis (553 cases, 721 controls) found a strong association between *H. pylori* infection and IDA in pregnant women (OR: 16.2; 95%CI: 4.2-62.9; *P*<.01)^14^. A landmark meta-analysis^15^ including pediatric and adult studies (with a ratio of two pediatric studies for every one adult study) revealed an overall association between *H. pylori* and IDA (OR: 1.7; 95%CI: 1.2-2.4). Subgroup analysis showed a stronger association in children (OR: 2.2; 95%CI: 1.4-3.4) than in adults (OR: 1.7; 95%CI: 1.0-2.9). The association with iron deficiency alone was similar across age groups, with an OR of 1.4 (95%CI: 1.2-1.8) for children and an OR of 1.4 (95%CI: 1.0-1.9) for adults^15^.

Another meta-analysis focusing on children (942 *H. pylori*-positive and 1,863 healthy controls) indicated an increased risk of developing IDA in infected children compared with uninfected children (OR: 1.8; 95%CI: 1.4-2.4) in prospective cohorts^16^. Case-control studies within the same review showed an even stronger association (OR: 3.8; 95%CI: 1.8-8.1)^16^. This study also found lower Hb and ferritin levels in infected children^16^.

These results raise critical questions: how *H. pylori*-associated IDA should be managed? Is eradication alone sufficient to correct the anemia? These questions were indirectly addressed by a recent meta-analysis of 23 studies^17^. *H. pylori* eradication increased Hb levels regardless of iron supplementation, although the study populations were highly heterogeneous^17^. However, there was no significant increase in Hb levels when comparing iron supplementation alone with combined therapy (iron supplementation plus *H. pylori* eradication)^17^. These counterintuitive findings indicate the contribution of other factors to IDA, significant cohort heterogeneity, or insufficiently long follow-up periods in the studies^17^. For ferritin levels, however, *H. pylori* eradication showed a clear advantage over iron supplementation alone, while combining eradication with supplementation yielded no statistically significant difference compared with eradication alone^17^.

Consensus guidelines on the management of *H. pylori* in adults recognize the link between the infection and IDA, recommending *H. pylori* testing and eradication in patients with IDA as part of the anemia treatment strategy. Major hematology guidelines concur with this approach^18^ ^-^ ^23^.

Conversely, for children and adolescents, the joint European-North American guidelines do not recommend testing for *H. pylori* during the initial evaluation of IDA, even via noninvasive methods^24^. However, if an upper endoscopy is conducted during the diagnostic workup for IDA, *H. pylori* should be tested for and, if present, eradicated, in addition to initiating iron supplementation^24^.

In summary, robust evidence supports the association between IDA and *H. pylori* infection, particularly in children and pregnant women. In adults, the presence of IDA warrants screening and eradication of *H. pylori*, whereas this routine approach does not apply to children and adolescents. Finally, eradicating *H. pylori* appears to improve Hb and ferritin levels, particularly when accompanied by iron supplementation.

### Immune thrombocytopenia

ITP is an autoimmune hematologic disorder characterized by the destruction of peripheral blood platelets and impaired platelet production by bone marrow megakaryocytes^25^. The diagnosis of ITP is based on a platelet count <100 × 10^9^/L, and the condition is classified as newly diagnosed (duration <3 months), persistent (3 to 12 months), or chronic (>12 months)^26^. ITP can be a primary disease or secondary to various etiologies, including infections, autoimmune diseases, or malignancies. A chronic inflammatory state following a primary bacterial or viral infection can trigger a host autoimmune response via multiple mechanisms, including autoantibody production and immune complex formation^27^. The incidence of ITP is approximately 2.7 per 100,000 in adults and 5 per 100,000 in children, with a female-to-male ratio of 1.7:1^28^.

The first evidence linking ITP to *H. pylori* infection was published in 1998 by Gasbarrini et al., who reported an increase in platelet counts in all 8 patients with ITP in whom *H. pylori* was successfully eradicated. Conversely, platelet counts remained low in 3 patients in whom eradication therapy failed and in 7 *H. pylori*-negative patients^29^.

The exact mechanisms underlying the role of *H. pylori* in the pathophysiology of ITP remain incompletely understood. Molecular mimicry is the most widely accepted theory, suggesting that antibodies induced by *H. pylori* cross-react with platelet surface antigens, leading to their destruction^27^ ^,^ ^30^. Molecular mimicry between CagA antigens and platelet surface glycoproteins, leading to the production of platelet-associated IgG (PAIgG) antibodies-which play a well-known role in ITP pathogenesis-has been extensively documented^31^. Another proposed mechanism is the direct binding of *H. pylori* antigens, such as Lpp20, which can trigger platelet activation and aggregation, ultimately causing platelet destruction^30^ ^,^ ^32^ ^,^ ^33^. Furthermore, thrombocytopenia could result from the host’s immune response, characterized by the inhibition of Fcγ receptors on monocytes and the expansion of dendritic cells producing IL -10 and IL-12^34^ ^,^ ^35^.

Most studies investigating the link between *H. pylori* and ITP are retrospective, nonrandomized, or randomized but with small sample sizes. A systematic review of 25 studies involving 1,555 patients with ITP reported a complete response rate of 42.7% (95%CI: 31.8%-53.9%) among those who underwent successful *H. pylori* eradication. Regression analysis revealed a significant positive correlation between the prevalence of *H. pylori* infection in cohorts of patients with ITP and the platelet response to eradication therapy^36^. However, when the analysis was restricted to patients with severe thrombocytopenia (platelet count <30,000/µL; 15 studies, 222 patients with eradicated *H. pylori*), the complete response rate dropped to 20.1% (95%CI: 13.5%-26.7%), and the overall response rate fell to 35.2% (95%CI: 28.0%-42.4%). This indicates that outcomes are superior in patients with ITP who have milder thrombocytopenia^36^. Across studies, platelet response rates were higher in regions where the population prevalence of *H. pylori* infection is high^27^ ^,^ ^36^ ^,^ ^37^. A 2018 study conducted in California also suggested ethnic and racial differences, noting higher platelet response rates following eradication in Hispanic and Asian populations^38^.

In the pediatric population, the platelet response to *H. pylori* eradication is less robust than in adults. Consequently, the recent ESPGHAN/NASPGHAN guidelines revised their prior stance and no longer recommend routine *H. pylori* testing in children with ITP^24^. The 2019 International Consensus Report on ITP assigned a Grade C recommendation for testing for *H. pylori* in the pediatric population, but a Grade B recommendation (Level 2A evidence) for its inclusion in the initial diagnostic workup of adults with ITP^39^. The Fourth Brazilian Consensus on *H. pylori* Infection states there is evidence connecting the infection to ITP (Grade B recommendation, Level 3A evidence), and the Maastricht Consensus recommends eradicating *H. pylori* in patients with ITP^18^ ^,^ ^19^.

In summary, a positive correlation exists between ITP and *H. pylori* infection, and platelet levels improve following eradication-particularly in adults, patients with milder degrees of thrombocytopenia, and those from areas with a high incidence of *H. pylori* infection.

### Colorectal cancer

Investigations into the relationship between *H. pylori* infection and CRC began in the early 2000s. The first study to directly highlight this link was published by Shmuely et al. in 2001, demonstrating that patients infected with CagA-positive strains of *H. pylori* had a significantly higher risk of colorectal adenocarcinoma (OR: 10.6; 95%CI: 2.7-41.3; *P*=.001) compared with individuals infected with CagA-negative strains^40^. Since then, numerous studies have sought to confirm this association, albeit with conflicting results.

Observational studies, meta-analyses, and systematic reviews indicate an increased risk of adenomatous polyps and CRC in *H. pylori*-infected individuals, with ORs ranging from 1.36 to 1.80 across different populations^41^ ^-^ ^43^. In analyses conducted in East Asian populations, particularly in China, this risk was significant (OR: 1.58; 95%CI: 1.05-2.37)^43^. Conversely, studies in other East Asian nations, such as Japan and South Korea, did not find a statistically significant association (OR: 1.26; 95%CI: 0.93-1.70)^44^. European studies have also suggested a higher likelihood of CRC in individuals seropositive for specific *H. pylori* proteins, such as HcpC and VacA^44^.

Despite this evidence, it remains unclear whether a causal relationship exists. Mendelian randomization studies-which minimize confounding bias-have failed to establish a robust causal link between the infection and CRC development^42^. Similarly, large population-based cohorts, such as one conducted in Sweden, did not show a consistent risk reduction following bacterial eradication, implying that the observed effects may stem from indirect or confounding factors^45^.

A retrospective cohort study by Shah et al. evaluated the relationship between *H. pylori* infection, treatment, and CRC risk in a large U.S. veteran population^46^. *H. pylori* positivity was associated with an 18% higher risk of incident CRC and a 12% higher risk of CRC-related mortality^46^. Being *H. pylori*-positive versus *H. pylori*-negative was associated with an 18% (adjusted hazard ratio [adjusted HR], 1.18 [95%CI, 1.12 to 1.24]) and 12% (adjusted HR, 1.12 [95%CI, 1.03 to 1.21]) higher incident and fatal CRC risk, respectively. These findings indicate that *H. pylori* infection may contribute to a modest but significant increase in CRC risk, and that treatment is associated with mitigating this risk^46^.

Evidence from animal models is growing, though it remains limited and heterogeneous. A study using mice with Apc gene mutations found that *H. pylori* infection was linked to an increased tumor burden and earlier onset of lesions^47^. Immunological changes were also observed, including increased Th17 cell populations, decreased regulatory T cell counts, STAT3 pathway activation, reduced goblet cell counts, and shifts in the gut microbiota favoring mucus-degrading species. Eradicating *H. pylori* lowered tumor incidence to levels seen in uninfected animals, pointing to a causal role for the bacterium and indicating that its elimination could serve as a preventive measure against CRC^47^.

The proposed pathophysiological mechanisms linking *H. pylori* infection to CRC development involve multiple interconnected pathways, most notably gut microbiota modulation, induction of local and systemic inflammation, and the effects of specific bacterial factors^48^ ^-^ ^50^. Animal and human studies demonstrate that *H. pylori* strains, particularly those carrying the cagA gene, promote intestinal dysbiosis marked by reduced bacterial diversity and an overgrowth of potentially pathogenic microorganisms, such as Staphylococcus and Corynebacterium^48^. This microbial shift compromises mucosal barrier integrity, facilitating bacterial translocation and activating chronic inflammatory responses in the colon-events well documented in colorectal carcinogenesis^48^ ^-^ ^50^.

Additionally, *H. pylori* infection raises both local and systemic levels of proinflammatory cytokines, such as TNF-α, IL-6, and IL-8, while increasing the expression of major histocompatibility complex class II (MHC II) on antigen-presenting cells. This creates an inflammatory microenvironment highly conducive to neoplastic transformation^48^ ^,^ ^49^. Another key mechanism is the direct action of bacterial toxins such as VacA and CagA, which modulate the immune response and induce colonic epithelial changes; serological studies have linked these toxins to a higher risk of colorectal neoplasia^48^.

To date, no formal guidelines recommend a specific CRC screening methods based solely on *H. pylori* status. Major medical societies advise CRC screening based on classical risk factors (age, family history, inflammatory bowel disease, hereditary syndromes), utilizing modalities such as colonoscopy, fecal immunochemical testing (FIT), flexible sigmoidoscopy, stool DNA testing, and CT colonography, irrespective of *H. pylori* infection^51^.

Coelho et al. reviewed the association between *H. pylori* and CRC through the lens of Sir Austin Bradford Hill’s criteria for causality^52^. Their analysis of observational studies, clinical trials, and meta-analyses showed a modest but statistically significant positive association. The temporality criterion is strongly met, as infection typically occurs in childhood, preceding CRC onset by decades. A biological gradient was observed, with a greater risk linked to virulent strains (CagA+, VacA+) and premalignant gastric lesions. Biological plausibility is supported by evidence that *H. pylori* triggers intestinal dysbiosis, chronic inflammation, oncogenic pathway activation, and genetic instability-all recognized mechanisms in colorectal carcinogenesis. Murine models confirmed that the infection accelerates tumor formation, and eradicating the bacterium reversed these pro-tumorigenic changes, thereby satisfying the experimental evidence criterion. Although specificity is low, the criteria for consistency, coherence, and analogy were reasonably met. Thus, the authors concluded that *H. pylori* adequately fulfills Bradford Hill’s criteria for causality. However, they highlighted that the strength of the association is modest and causality is not unequivocal, underscoring the need for further research. In conclusion, while epidemiological evidence links *H. pylori* to CRC, a direct causal relationship remains debated due to divergent findings across observational, genetic, and interventional studies. Geographic heterogeneity, methodological differences, and potential confounding variables complicate the establishment of a definitive consensus. This topic remains under active investigation, and clinical recommendations for *H. pylori* screening or eradication specifically to prevent CRC are not yet backed by robust, universal evidence.

### Cardiovascular diseases

CVD, particularly of ischemic origin, is the leading cause of morbidity and mortality worldwide^53^ ^-^ ^55^. In 2021, an estimated 21.5 million people died from CVD, accounting for roughly one-third of all global deaths^55^. Atherosclerosis is the primary pathophysiological mechanism underlying most of these conditions. While traditional risk factors-such as hypertension, dyslipidemia, diabetes mellitus, smoking, and obesity-account for about 50% to 60% of incident CVD events and mortality, this proportion suggests the existence of additional, not fully elucidated determinants^56^. In this context, emerging risk factors, including chronic infections, nano- and microplastics, pollution, microbiome alterations, and social determinants of health, are increasingly recognized as potential contributors to CVD onset and progression^57^ ^,^ ^58^.

Among these, although its exact role remains controversial, *H. pylori* infection has been linked to elevated cardiovascular risk and is detected in approximately 56.7% of patients with CVD^59^ ^,^ ^60^. Several meta-analyses, cohort studies, and cross-sectional studies indicate that *H. pylori* infection is associated with an increased risk of CVD, coronary artery disease, and stroke, particularly in individuals harboring CagA-positive strains^60^ ^-^ ^64^. In a comprehensive meta-analysis of 44 studies, the presence of anti-*H. pylori* IgG antibodies correlated with a higher risk of coronary artery disease (OR: 1.58; 95%CI: 1.34-1.87)^63^. Similarly, seropositivity for anti-CagA antibodies, fecal antigen positivity, and histological detection of *H. pylori* also showed significant associations with coronary artery disease, yielding ORs of 1.33 (95%CI: 1.16-1.53), 3.50 (95%CI: 1.60-7.66), and 1.78 (95%CI: 1.12-2.83), respectively^63^.

Additionally, the detection of Helicobacter spp. DNA in the atherosclerotic plaques of patients with advanced coronary artery disease bolsters the hypothesis that the infection may influence atherosclerosis development^65^. Assessments of carotid intima-media thickness suggest that the infection may be associated with arterial wall thickening-a subclinical marker of atherosclerosis-and increased arterial stiffness^66^ ^-^ ^69^. In a propensity score-matched analysis of a prospective study involving 4,765 *H. pylori*-infected patients with a median follow-up of 51 months, bacterial eradication significantly reduced the risk of coronary artery disease in men aged ≤65 years (HR: 0.133; 95%CI: 0.039-0.455; *P*=.001) and in women aged >65 years (HR: 0.260; 95%CI: 0.110-0.615; *P*=.002)^70^. Finally, Doheim et al. demonstrated an increased risk of ischemic stroke in patients with positive *H. pylori* serology (OR: 1.43; 95%CI: 1.25-1.46), a finding that was also significant for anti-CagA antibodies (OR: 1.77; 95%CI: 1.25-2.49) and a positive C-urea breath test (OR: 2.21; 95%CI: 1.33-3.66)^61^.

### Proposed pathogenic mechanisms

The potential link between *H. pylori* and CVD is based on several plausible pathophysiological mechanisms:

**Systemic chronic inflammation:** *H. pylori* infection triggers a sustained systemic inflammatory response, elevating proinflammatory cytokines such as IL-1β, IL-6, IL-8, and TNF-α. These mediators drive endothelial activation, expression of adhesion molecules (ICAM-1, VCAM-1), leukocyte recruitment, and vascular damage-all central processes in atherogenesis. Moreover, infection-induced oxidative stress promotes the oxidation of LDL particles, which are then phagocytosed by macrophages via scavenger receptors, turning them into foam cells and accelerating plaque formation^67^ ^,^ ^71^. Molecular mimicry between *H. pylori* and host components, leading to the production of cross-reactive antibodies, is also suggested as a contributing mechanism^72^.

**CagA-mediated endothelial dysfunction:** The CagA protein, released in exosomes by virulent *H. pylori* strains, can enter the systemic circulation and be internalized by macrophages in the arterial wall^73^. This protein inhibits the transcription factors PPARγ and LXRα, impairing the expression of ABCA1, ABCG1, and SR-BI transporters, which are responsible for cholesterol efflux. This promotes intracellular lipid accumulation and foam cell formation^74^. Concurrently, CagA upregulates COX-1 and COX-2 expression, driving the synthesis of thromboxane A2 and prostaglandin E2, which contribute to platelet aggregation and vasoconstriction^75^.

**Hyperhomocysteinemia:** Chronic infection can lead to gastric atrophy and vitamin B12 deficiency, resulting in the inhibition of methionine synthase and elevated homocysteine-a well-known risk factor for endothelial dysfunction and CVD^76^ ^-^ ^78^.

**Metabolic alterations:** *H. pylori* may disrupt lipid metabolism, lowering HDL and raising LDL levels^79^. One meta-analysis also found an association between the infection and a higher risk of hypertension (OR: 1.34; 95%CI: 1.10-1.63; *P*=.002)^80^. Other studies indicate improvements in glycemic control following bacterial eradication^81^.

### Controversies and perspectives

Despite mechanistic and associative evidence, the link between *H. pylori* and CVD remains controversial. Discrepancies may stem from heterogeneous diagnostic methods (serology, stool antigen, breath test, histology), confounding by established CVD risk factors, and geographic differences in strain incidence and virulence. Currently, there is no formal recommendation to eradicate *H. pylori* for the purpose of CVD prevention. Nevertheless, its elimination has been shown to reduce systemic inflammatory markers in some studies, hinting at a potential indirect benefit. Importantly, eradication is highly relevant in patients on antiplatelet therapy, as it significantly reduces the risk of ulcer bleeding (HR: 0.35; 95%CI: 0.14-0.89; *P*=.028)^82^.

### Neurological diseases

Neurological conditions have also been linked to *H. pylori* infection. It is hypothesized that the presence of the bacterium may activate systemic inflammatory pathways capable of disrupting the microbiota-gut-brain axis and promoting neurodegeneration, as well as metabolic and vascular dysfunction.

### Dementia and alzheimer disease

A 2016 systematic review analyzed seven observational studies evaluating the association between *H. pylori* infection and the onset of dementia, identifying a positive association between the two (OR: 1.71; 95%CI: 1.17-2.49; *P*=.01). However, upon subgroup analysis, the association lost statistical significance when stratified by dementia subtype (Alzheimer disease vs. non-Alzheimer dementia). In the context of dementia, the proposed pathogenic mechanisms include chronic inflammation, deficiencies in vitamin B12 and iron, elevated homocysteine, and the infection’s link to diabetes-all established risk factors for dementia^83^. A subsequent meta-analysis of observational studies also noted a positive correlation between *H. pylori* infection and dementia. Pooled cohort studies showed a significant association between *H. pylori* infection and all-cause dementia, yielding a relative risk (RR) of 1.36 (95%CI: 1.11-1.67). However, no significant association was found between *H. pylori* infection and the specific risk of developing Alzheimer disease [cohort studies: RR: 1.33 (95%CI: 0.86-2.05); case-control studies: OR: 1.72 (95%CI: 0.97-3.04)]^84^.

Thus, current studies suggest a modest association between *H. pylori* infection and the risk of all-cause dementia, but to date, this link has not been confirmed specifically for Alzheimer disease.

### Parkinson disease (PD)

The literature regarding the association between *H. pylori* and PD remains conflicting. A 2018 meta-analysis assessed the prevalence of *H. pylori* in patients with PD, examining the link between the infection and both motor symptom severity and post-eradication motor outcomes. The results demonstrated a higher prevalence of *H. pylori* infection in patients with PD (OR: 1.47; 95%CI: 1.27-1.70). Infected patients had significantly higher motor severity scores compared with uninfected patients with PD, and there was a partial improvement in the patients’ motor symptom scores following bacterial eradication^85^. However, a 2025 single-center Chinese case-control study found that patients with PD exhibited a significantly lower prevalence of *H. pylori* infection, even after adjusting for confounding factors^86^. A 2021 systematic review evaluated the available evidence regarding the potential benefits of *H. pylori* eradication in PD, finding that it could improve gait and motor symptom severity scores^87^. A study published the same year investigated the interaction between *H. pylori* infection and the pharmacokinetics of levodopa therapy in PD. The authors suggested that the clinical benefits of eradication might stem from improved levodopa absorption and bioavailability; however, they did not recommend universal screening or treatment of the infection solely to improve parkinsonian symptoms^88^.

### Stroke

Several studies indicate that *H. pylori* infection is associated with an increased risk of stroke, particularly of atherosclerotic origin, pointing to the bacterium’s role in atherogenesis and endothelial dysfunction, as demonstrated in the meta-analysis by Doheim et al.^61^. International guidelines have recognized a potential link between these conditions since 2006, highlighting a greater risk tied to CagA-positive strains^89^. The evidence, however, is not yet sufficient to recommend eradication as a primary prevention measure for stroke.

### Multiple sclerosis

The most recent and robust evidence indicates no significant association between *H. pylori* infection and the risk of developing multiple sclerosis. Therefore, *H. pylori* is considered neither a risk nor a protective factor for the disease^90^.

### Concluding remarks

In this review, we synthesized recent evidence examining the association between H. pylori infection and a broad spectrum of extra-gastric conditions. Several recent studies deserve particular attention. A 2025 meta-analysis evaluating the impact of H. pylori infection on iron-deficiency anemia in children provided updated quantitative evidence supporting an increased risk in infected pediatric populations, with odds ratios ranging from 1.8 to 3.8^15^.

Increasing evidence also suggests a potential link between H. pylori infection and colorectal cancer (CRC). A retrospective study reported an 18% increased risk of CRC incidence and a 12% higher CRC-related mortality among H. pylori-positive individuals^46^. Importantly, experimental evidence from an animal model demonstrated that bacterial eradication reversed pro-tumorigenic alterations, supporting a causal relationship according to the Bradford Hill criteria for colorectal carcinogenesis^47^.

In contrast, the association with Parkinson’s disease remains controversial. A case-control study conducted in China and published in 2025 reported a significantly lower prevalence of H. pylori infection among patients with Parkinson’s disease, challenging earlier meta-analyses from 2018 that suggested a positive association^86^. These conflicting findings highlight the complexity of the interaction between chronic infection, neuroinflammation, and neurodegenerative processes.

Mechanistic insights have also advanced in the field of cardiovascular disease. Recent studies suggest that the CagA protein can be released in exosomes and enter the systemic circulation, promoting foam cell formation within arterial walls and accelerating atherosclerosis. These discoveries strengthen the biological plausibility of a link between H. pylori infection and cardiovascular disease^73^ ^-^ ^75^.

Taken together, these findings suggest that the conceptual framework surrounding H. pylori infection is evolving-from a pathogen primarily associated with gastric disorders to a potential systemic contributor to multiple extra-gastric diseases. Recognizing this broader pathogenic role may open new avenues for research and could ultimately influence future clinical strategies.

## CONCLUSION

Historically, research on *H. pylori* has progressed through the resolution of major scientific controversies. The recognition of this microorganism as the etiologic agent of peptic ulcer disease became widely accepted only after substantial evidence confirmed the pioneering observations of Warren and Marshall^91^, ultimately overcoming initial skepticism within the medical community. More than four decades after its original description, new research frontiers continue to emerge. The current challenge is to determine whether the reported associations between *H. pylori* and extragastric conditions represent mere statistical correlations or whether they carry genuine clinical significance-adding yet another compelling chapter to this remarkable scientific narrative.

## Data Availability

Data availability statement: Not applicable
